# Treatment of Posttraumatic Stress Disorder Using the Traditional Japanese Herbal Medicine Saikokeishikankyoto: A Randomized, Observer-Blinded, Controlled Trial in Survivors of the Great East Japan Earthquake and Tsunami

**DOI:** 10.1155/2014/683293

**Published:** 2014-03-24

**Authors:** Takehiro Numata, Shen GunFan, Shin Takayama, Satomi Takahashi, Yasutake Monma, Soichiro Kaneko, Hitoshi Kuroda, Junichi Tanaka, Seiki Kanemura, Masayuki Nara, Yutaka Kagaya, Tadashi Ishii, Nobuo Yaegashi, Masahiro Kohzuki, Koh Iwasaki

**Affiliations:** ^1^Department of Obstetrics and Gynecology, Tohoku University Graduate School of Medicine, No. 1-1, Seiryo-Machi, Aoba Ward, Sendai City 980-8574, Japan; ^2^Department of Internal Medicine and Rehabilitation Science, Tohoku University Graduate School of Medicine, No. 1-1, Seiryo-Machi, Aoba Ward, Sendai City 980-8574, Japan; ^3^Comprehensive Education Center for Community Medicine, Tohoku University Graduate School of Medicine, No. 2-1, Seiryo-Machi, Aoba Ward, Sendai City 980-8575, Japan; ^4^Center for Traditional Asian Medicine, National Sendai-Nishitaga Hospital, No. 2-11-11, Kagitorihoncho, Taihaku Ward, Sendai City 982-8555, Japan; ^5^Graduate Medical Education Center, Tohoku University Hospital, No. 1-1, Seiryo-Machi, Aoba Ward, Sendai City 980-8574, Japan; ^6^Division of General Medicine, Saitama Medical Center, Jichi Medical University, No. 1-847, Amanumacho, Oomiya Ward, Saitama City 330-8503, Japan; ^7^Department of Education and Support for Community Medicine, Tohoku University Hospital, No. 1-1, Seiryo-Machi, Aoba Ward, Sendai City 980-8574, Japan

## Abstract

The Great East Japan earthquake and tsunami caused immense damage over a wide area of eastern Japan. Hence, many survivors are at high risk for posttraumatic stress disorder (PTSD). This randomized, observer-blinded, controlled trial examined the efficacy and safety of the traditional Japanese herbal formula saikokeishikankyoto (SKK) in the treatment of PTSD among survivors of this disaster. Forty-three participants with an Impact of Event Scale-Revised (IES-R) score ≥ 25 were randomized into SKK (*n* = 21) and control (*n* = 22) groups. The primary endpoint was the change in IES-R scores from baseline till after 2 weeks of treatment. Intergroup statistical comparisons were performed. The magnitude of changes in total IES-R scores differed significantly between the two groups (*P* < 0.001). Post hoc analysis showed that the total IES-R score improved significantly in the SKK group from 49.6 ± 11.9 to 25.5 ± 17.0 (*P* < 0.001). Subscale scores improved significantly in the SKK group (avoidance, *P* = 0.003; hyperarousal, *P* < 0.001; intrusion, *P* < 0.001). Two-week treatment with SKK significantly improved IES-R scores among PTSD patients. This traditional medicine may be a valid choice for the treatment of psychological and physical symptoms in PTSD patients.

## 1. Introduction

Two years have passed since March 11, 2011, the day the Great East Japan earthquake and tsunami claimed 20,000 lives in a flash. Although people have been able to move from shelters to temporary housing, heaps of rubble and radioactive pollution prevent the rehabilitation of many areas. While some commercial fishing has resumed, inshore fish are polluted with radiation and cannot be sold. The towns and cities inundated by the tsunami are, even now, a wasteland. The disaster caused posttraumatic stress disorder (PTSD) in many people [1]. Many patients have visited our clinic for PTSD treatment, but psychotherapists are in short supply. We attempted to treat them using mood stabilizers, antidepressants, and hypnotics, but these medications are not always effective; some of these patients then suffer from intractable drug dependency. A Chinese herbal formula was reported to be effective for patients with PTSD after the Sichuan earthquake in 2008 [2]. Saikokeishikankyoto (SKK, Chaihu-Guizhi-Ganjiang-Tang in Chinese) is a traditional Japanese-Chinese herbal formula that has a marked effect on some PTSD patients. We therefore conducted a randomized observer-blinded trial of the efficacy and safety of SKK for the treatment of PTSD.

## 2. Methods

### 2.1. Participants

Subjects were recruited from the outpatient clinic at the National Sendai-Nishitaga Hospital in Sendai, Japan. The required sample size was calculated as 20 subjects for each arm, with an alpha error of 0.05 and power of 0.8. All participants completed an intake assessment that included medical history, physical examination, and standard blood examination as well as an Impact of Event Scale-Revised score (IES-R) (see the following pattern) to assess the severity of PTSD at baseline.

*Scoring the Impact of Event Scale-Revised Questionnaire*

*Impact of Event Scale-Revised Questionnaire*

*Instructions*. Below is a list of difficulties people sometimes have after stressful life events. Please read each item, and then indicate how distressing each difficulty has been for you during the past seven days with respect to _________________________, which occurred on ______________. How much were you distressed or bothered by these difficulties?

Item Response Anchors are as follows: 0 = not at all; 1 = a little bit; 2 = moderately; 3 = quite a bit; 4 = extremely.

The Intrusion subscale is the mean item response of items 1, 2, 3, 6, 9, 14, 16, and 20. Thus, scores can range from 0 to 4. The avoidance subscale is the mean item response of items 5, 7, 8, 11, 12, 13, 17, and 22. Thus, scores can range from 0 to 4. The hyperarousal subscale is the mean item response of items 4, 10, 15, 18, 19, and 21. Thus, scores can range from 0 to 4. Consider the following.

1. Any reminder brought back feelings about it.
2. I had trouble staying asleep.
3. Other things kept making me think about it.
4. I felt irritable and angry.
5. I avoided letting myself get upset when I thought about it or was reminded of it.
6. I thought about it when I did not mean to.
7. I felt as if it had not happened or was not real.
8. I stayed away from reminders of it.
9. Pictures about it popped into my mind.
10. I was jumpy and easily startled.
11. I tried not to think about it.
12. I was aware that I still had a lot of feelings about it, but I did not deal with them.
13. My feelings about it were kind of numb.
14. I found myself acting or feeling like I was back at that time.
15. I had trouble falling asleep.
16. I had waves of strong feelings about it.
17. I tried to remove it from my memory.
18. I had trouble concentrating.
19. Reminders of it caused me to have physical reactions, such as sweating, trouble breathing, nausea, or a pounding heart.
20. I had dreams about it.
21. I felt watchful and on-guard.
22. I tried not to talk about it.

Total IES-R score: _____________.

The inclusion criteria were as follows: (1) survivors of the Great East Japan earthquake and tsunami who were older than 20 years and diagnosed with PTSD according to the Diagnostic and Statistical Manual (DSM)-IV TR; (2) IES-R score ≥ 25 (cutoff point).

Exclusion criteria were as follows: (1) major medical illness such as neoplastic disease, acute inflammation, or any other disease that would most likely prevent the completion of this study; (2) psychosis due to other disorders such as schizophrenia, depression, and/or dementia; (3) delirium due to drugs, alcohol, metabolic intoxication, or inflammation; and (4) use of neuroleptics, antianxiety drugs, antiepileptic drugs, antidepressants, or herbal remedies during the past 2 months.

Participants underwent a complete diagnostic assessment, including medical history, physical examination, laboratory tests, and the Structured Clinical Interview for DSM-IV TR and IES-R. Eligible subjects were randomized into either the SKK or control group. Random numbers were generated using computer software. Treatment codes were held by the corresponding investigator, who was isolated from the patients and outcome data.

The study protocol was performed with the intention to treat. SKK extract was prescribed to the patients in the SKK group. The SKK extract (TJ-11) was processed by Tsumura (Tokyo, Japan), and it contained the following mixture of dried herbs: Bupleuri Radix (6 g), Trichosanthis Kirilowii (3 g), Cinnamomi Cassiae (3 g), Radix Scutellariae Baicalensis (3 g), Concha Ostreae (3 g), Glycyrrhizae Radix (2 g), and dried Zingiberis Rhizoma (2 g). These herbs are registered in the Pharmacopoeia of Japan version 15. Each participant in the SKK group received 2.5 g of SKK powder (1.17 g extract) 3 times a day for 2 weeks. The processes involved in the production and supply of SKKcomply with Good Manufacturing Practices for Kampo products and are also approved by the Ministry of Health, Labour, and Welfare of Japan.

Participants understood that those randomized into the control group could receive any treatment after completion of the whole trial if they wanted. Patients were free to withdraw at any time. Clinical assessments were performed at the baseline and at the endpoint.

The primary clinical outcome measure was the severity of PTSD symptoms as measured by the total IES-R. The secondary outcome measures were 3 IES-R subscale scores as defined below. The intrusion subscale is the mean response to items 1, 2, 3, 6, 9, 14, 16, and 20. The avoidance subscale is the mean response to items 5, 7, 8, 11, 12, 13, 17, and 22. The hyperarousal subscale is the mean response to items 4, 10, 15, 18, 19, and 21.

### 2.2. Statistical Analysis

Analyses were performed as a modified intention to treat. If a patient dropped out, all outcome measures were assessed within a week. Statistical analysis was performed using the SPSS software (version 16, SPSS Japan Inc., Tokyo, Japan). Measurements of the mean and SD were calculated at the baseline and at the endpoint for all continuous primary and secondary measures. Baseline comparisons of group differences were conducted using independent samples* t*-tests for continuous variables and chi-square test for categorical variables. Comparisons between the SKK and the control group were performed by the two-way analysis of variance (ANOVA). The changes in each group from baseline to endpoint were compared using the paired* t*-test when the intergroup difference was significant (*P* < 0.05) according to the post hoc test.

This study was carried out in compliance with the ethical principles embodied in the Helsinki Declaration. Written informed consent was obtained from each participant prior to participation in this study. The study protocol was approved by the Institutional Review Board of Sendai-Nishitaga National Hospital in Sendai, Japan, and registered with the UMIN clinical trial registry (UMIN000010890, http://www.umin.ac.jp/ctr/index.htm).

## 3. Results

Of the 48 enrolled subjects, 5 were excluded according to the exclusion criteria. The remaining 43 participants were randomized into SKK (*n* = 21) and control (*n* = 22) groups. The background factors for each group are shown in Table 1, with no significant difference except for the IES-R hyperarousal subscale score. One participant in the SKK group withdrew from the study on the third day because of coughing; all participants in the control group completed the study.

### 3.1. Changes in Total IES-R Scores

**Figure 1:**
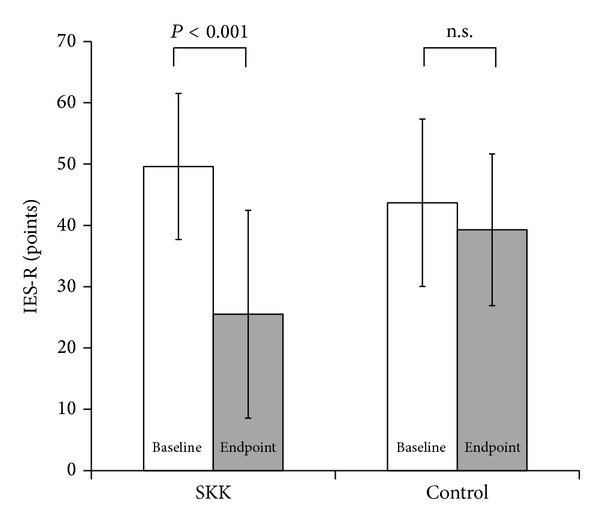
Changes in the total IES-R scores. The two-way analysis of variance (ANOVA) showed a significant difference between the groups (*P* < 0.001) and post hoc testing showed that total IES-R scores were significantly improved only in the SKK group.

Two-way ANOVA analysis showed that the changes in total IES-R scores differed significantly between groups (*P* < 0.001). The post hoc testing showed that the total IES-R score was significantly improved from 49.6 ± 11.9 to 25.5 ± 17.0 in the SKK group (*P* < 0.001) but decreased from 43.7 ± 13.7 to 39.3 ± 12.4 in the control group. The latter trend was not significant (Figure 1).

### 3.2. Changes in IES-R Subscale Scores

**Figure 2:**
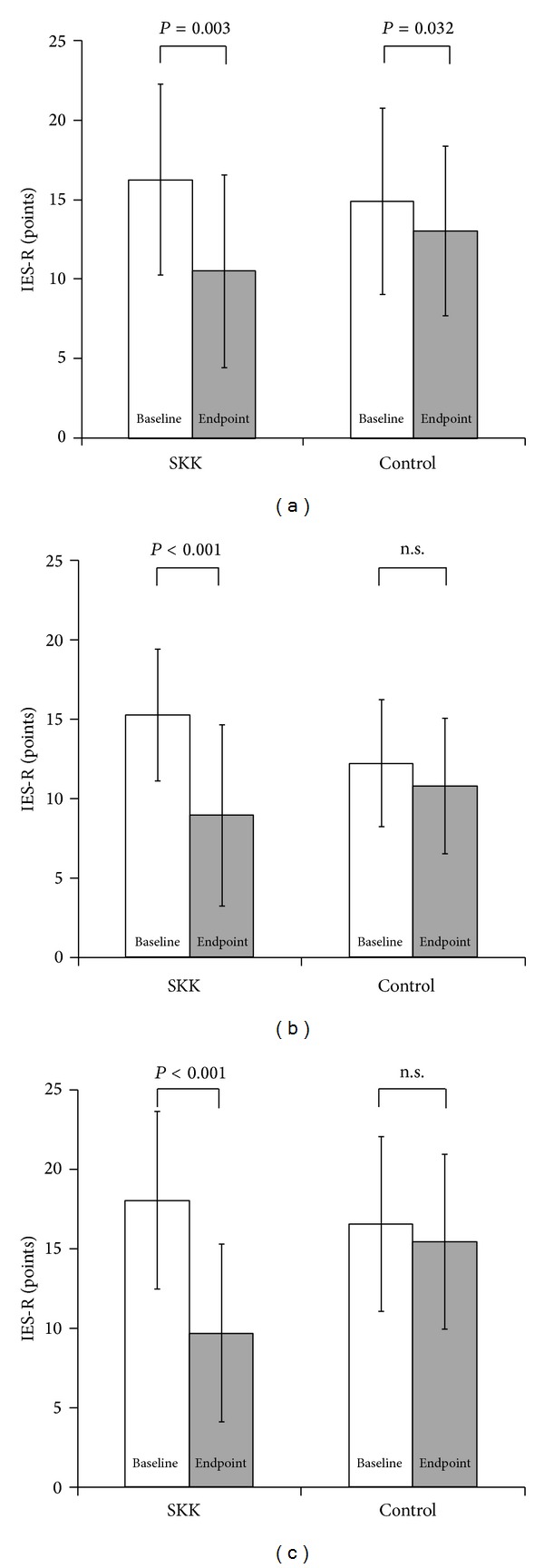
(a) Change in the IES-R avoidance subscale score. (b) Change in the IES-R hyperarousal subscale score. (c) Change in the IES-R intrusion subscale score. Two-way ANOVA showed that each subscale differed significantly between the groups ((a) *P* = 0.025, (b) *P* = 0.005, (c) *P* = 0.001). Post hoc testing showed that all subscale scores changed significantly from baseline to the endpoint in the SKK group ((a) *P* = 0.003, (b) *P* < 0.001, and (c) *P* < 0.001), whereas in the control group, only the avoidance subscale score showed a significant change ((a) *P* = 0.032, (b) n.s., and (c) n.s.).

The changes in IES-R subscale scores are shown in Figures 2(a), 2(b), and 2(c). Two-way ANOVA showed significant differences between groups (*P* = 0.025 for avoidance, *P* = 0.005 for hyperarousal, and *P* = 0.001 for intrusion), although there were baseline differences in scores on the hyperarousal subscale. The post hoc testing showed that all subscales improved significantly from baseline to the endpoint in the SKK group ((a) *P* = 0.003, (b) *P* < 0.001, and (c) *P* < 0.001), whereas only avoidance showed a significant change in the control group ((a) *P* = 0.032, (b) n.s., and (c) n.s.).

### 3.3. Significant Improvements in Each Item in the IES-R

The IES-R items that differed significantly between groups were as follows: “any reminder brought back feelings about it” (Q1, *P* < 0.001); “other things kept making me think about it” (Q3, *P* = 0.005); “I thought about it when I did not mean to” (Q6, *P* < 0.001); “I found myself acting or feeling like I was back in that time” (Q14, *P* = 0.003); “reminders of it caused me to have physical reactions, such as sweating, trouble breathing, nausea, or a pounding heart” (Q19, *P* = 0.001); “I had dreams about it” (Q20, *P* = 0.002); and “I felt watchful and on-guard” (Q21, *P* = 0.001).

### 3.4. Adverse Events and Follow-Up

No participant showed any abnormal change in the blood examination results. One participant in the SKK group withdrew from the study on the third day because of light coughing. The cough was minor and continued for 3 days. When he came back to the clinic 2 weeks later, he showed no symptoms or signs. The relationship between the treatment and the cough is unknown. After the trial, some participants in the control group were treated using traditional medicines according to the rules of the traditional Chinese-Japanese medical decision. They were treated with different medicines, including SKK. No systematic data are available.

## 4. Discussion 

The present study showed that SKK significantly improved PTSD caused by the disaster. A recent study showed that PTSD was strongly suspected in about 10% of all high school students in the city of Sendai at 9 months after the Great East Japan earthquake [3]. Both psychological and pharmacological treatments are effective for PTSD, but there is a severe shortage of psychologists and psychotherapists in the disaster-stricken area. General physicians and primary care doctors must routinely care for PTSD patients. Antidepressants, benzodiazepine, and antipsychotics are used to treat PTSD, but these medications have adverse effects. Drug dependency is common with the use of benzodiazepine. Selective serotonin reuptake inhibitors are also used to treat PTSD, but continuous treatment is often necessary to prevent relapse. Better pharmacological treatments that are both safe and effective are thus needed. Since the disaster, we have tried traditional herbal medicines, such as yokukansan (Yigan San), known to be effective for the treatment of mental disorders [4]. We observed that patients treated with SKK showed very clear improvements in PTSD symptoms. These clinical observations lead us to this trial. This study shows that SKK treatment resulted in the marked, rapid, and tolerable amelioration of PTSD symptoms in all participants, with no severe adverse events. In particular, SKK improved patient responses to the following items: “any reminder brought back feelings about it,” “other things kept making me think about it,” “I thought about it when I did not mean to,” “I found myself acting or feeling like I was back in that time,” “reminders of it caused me to have physical reactions, such as sweating, trouble breathing, nausea, or a pounding heart,” and “I felt watchful and on-guard.” These complaints may thus be indications for trying this medication. SKK also improved patient responses to “I had dreams about it.” Sleep disturbance is commonly resistant to treatment [5] and can lead to drug abuse [6].

Several pharmacological mechanisms underlying the effects of SKK have been investigated [7–9]. Acute moderate to high stress activates serotonergic neurons in the hippocampus to release 5-hydroxytryptamine (5HT). 5HT then activates postsynaptic 5HT1A receptors that inhibit the process of hippocampal long-term potentiation [7]. The repeated administration of SKK significantly increases the 5HT level in the hippocampus and the corpus striatum and of NE and 5HT in the hippocampus [8]. SKK also regulates plasma interleukin-6 and soluble interleukin-6 receptor concentrations and improves depressed mood in climacteric women with insomnia [9]. These findings may partially explain the mechanisms of SKK action in the treatment of mental disorders.

### 4.1. Limitations

The present study has some limitations. First, we could not include a placebo for comparison. Japanese law requires that if a placebo is used, it must be produced using the same procedures as the actual drug. Placebo preparation is thus impractical in Japan for a small pilot study like ours. Placebo effects may have influenced our data. The number of participants in this study was rather small but nonetheless reached the statistically targeted sample size. The observation time was short (14 days), because this was the maximum waiting time allowed for participants in the control group. No serious adverse events were observed in the present study, which is noteworthy when considering that Radix Scutellariae Baicalensis is suspected of causing interstitial pneumonia [10]; this relationship is unclear because incidents are rare. Hypokalemia caused by* Glycyrrhiza* root is well known, and it is observed in 6% of elderly subjects [11]. Clinical signs should be carefully monitored because SKK contains these herbs. More elaborate studies are required in the future to elucidate the effect and mechanism of SKK on patients.

Despite these limitations, the present study reports that the familiar herbal medicine SKK is significantly efficacious for the treatment of PTSD, with no serious adverse events. This information may be valuable for general physicians and primary care doctors who care for PTSD patients in Japan because SKK is a well-known traditional herbal medicine. The history of its clinical use in eastern countries dates back to 2 AD. We previously reported that traditional medicine was beneficial in the disaster-stricken area, where the modern medical system and its infrastructures, such as electricity, hospitals, clinics, and logistics, were entirely destroyed [12–14]. In such a situation, traditional medicine could be used to treat daily symptoms that manifest as physical findings among evacuees. Traditional medicine should be considered as a powerful tool for the practice of disaster medicine.

## 5. Conclusions

SKK significantly improved IES-R scores after 2 weeks of treatment. This traditional medicine may be a treatment choice for psychological and physical symptoms in PTSD patients.

## Figures and Tables

**Table 1:** Background factors for SKK and control groups.

	Group		*P* value
	SKK	Control	
*n*	21	22	
Sex (m/f)	9/12	13/9	0.45
Age (year)	52.3 ± 13.0	48.0 ± 20.9	0.42
IES-R (baseline)	49.6 ± 11.6	43.7 ± 13.7	0.14

## Abbreviations

PTSD: Posttraumatic stress disorder
IES-R: Impact of Event Scale-Revised score
DSM: Diagnostic and Statistical Manual
SKK: Saikokeishikankyoto
ANOVA: Analysis of variance.
